# The consistency of Federalist Society-affiliated U.S. supreme court justices

**DOI:** 10.1371/journal.pone.0329692

**Published:** 2025-08-18

**Authors:** Tim Komatsu, Paul M. Collins

**Affiliations:** 1 Department of Political Science, University of Massachusetts Amherst, Amherst, Massachusetts, United States of America; 2 Department of Legal Studies, University of Massachusetts Amherst, Amherst, Massachusetts, United States of America; National Taiwan University, TAIWAN

## Abstract

The U.S. Supreme Court has recently undergone a dramatic turn to the right, fundamentally reshaping law and politics in America. In this article, we examine one reason for this shift: the increasingly prominent role of the Federalist Society in the conservative legal movement. An analysis of almost 25,000 votes of Supreme Court justices from 1986–2022 shows that justices affiliated with the Federalist Society are about 10 percentage points more likely to cast a conservative vote than their non-affiliated counterparts, and the voting behavior of Federalist Society-affiliates is more ideologically consistent than non-affiliated justices. Because justices in the contemporary era serve on the Court for an average of about a quarter century, these findings indicate that we are likely to see the Court’s conservative justices – who are all Federalist Society-affiliates – continue to advance the conservative legal movement’s agenda for decades to come.

## Introduction

The conservative legal movement has substantially advanced its agenda in American law and politics, particularly at the U.S. Supreme Court. In recent years, the Court has embraced the goals of the movement on topics including reproductive freedom, gun control, voting rights, and business regulation. These decisions have fundamentally altered American society, while at the same time putting the movement and its actors in the spotlight of American political debate [1,2].

In this article, we examine one of the most timely and important questions about the success of the conservative legal movement: the extent to which Supreme Court justices connected to the movement exhibit behavior that is distinct from their counterparts. To do this, we investigate the voting behavior of justices affiliated with the Federalist Society, arguably the most important organization in the conservative legal movement [3–5]. We argue that Federalist Society-affiliated justices are more conservative than other justices, and are more *consistently* conservative, meaning that they cast votes that correspond to their conservative ideologies more often than other justices, even other conservative justices. By exhibiting this consistency, Federalist Society-affiliated justices push the conservative legal agenda over time, building precedents to support the movement’s goals. And, by being so consistent, they avoid the perceived mistakes of Republican-appointed justices that came before them who sometimes sided with more liberal justices to form majorities that undermined the goals of the movement [3].

This research is important for several reasons. We provide new insights into how the trainings and networking offered by the Federalist Society can pay dividends by generating consistency in its members, which translates into policy wins over time that advance the conservative agenda. We do this by showing how investigating the variability of judges’ decision making – and not just influences on the liberal or conservative nature of judicial votes – can help us better understand important public policy debates. Though our focus is on judges, the theoretical and methodological approaches we employ are applicable to a wide range of settings. For instance, the types of training and networking provided by the Federalist Society can also be found in non-governmental organizations [6], labor unions [7], and more. As such, this research can be applied beyond the legal system to understand the links between the goals of training and networking, and the behavior of actors. In addition, we advance our understanding of how lobbying operates and can succeed in a system in which actors are institutionally insulated from public and political pressure. In the case of American federal judges, this insulation is due to life tenure, but there are other ways to shield actors from political and public pressures, such as through merit-based civil service systems [8]. As we show, this type of insulation can motivate social movement actors to look for creative ways to influence behavior, including through education, networking, and tangible rewards. The existence and success of these novel lobbying approaches suggests the need to rethink the effectiveness of mechanisms intended to insulate actors from external pressures.

### The Federalist Society and judicial behavior

The Federalist Society for Law and Public Policy Studies was formed in 1982 with the goal “to provide an alternative to the perceived liberal orthodoxy” of the legal profession [3]. In addition, the Federalist Society established an important audience for conservative legal elites that is capable of holding them accountable for their actions and thus encouraging consistency in their decision making [3]. The Federalist Society differs from more traditional conservative public interest groups in that it does not participate in litigation or take positions on issues of public policy [9]. It is distinct from the American Bar Association in that it is not a professional organization of attorneys that sets academic standards, rates judges, and drafts model codes of ethics for lawyers. Instead, the Federalist Society promotes the goals of the conservative legal movement by performing three core, overlapping functions: education, networking, and advising [3,5,10].

The Federalist Society’s educational role is played in law schools, and also in the debates, conferences, publications, and other events hosted by the organization. There are currently Federalist Society chapters at every American Bar Association-affiliated law school [11]. These student chapters provide spaces for conservative law students to network with like-minded individuals, while also allowing the organization to court moderate and even liberal students [12]. Outside of law schools, the Federalist Society hosts forums and other events featuring legal heavyweights, including attorneys general, judges, and Supreme Court justices, in addition to creating educational content promoting the group’s goals intended for consumption by the general public [13]. Although many events hosted by the Federalist Society feature speakers from a diverse array of viewpoints, the organization is unabashedly devoted to promoting the goals of the conservative legal movement [3–5]. These goals include promoting the conservative judicial philosophy of originalism, traditional values, limited government, and overturning liberal Supreme Court precedents that are inconsistent with originalism [3,9,10,13].

The second core function of the Federalist Society is to serve as a network within the conservative legal movement. In this role, the organization is able to build off of its success at educating individuals to promote the goals of the movement by helping them build their networks in the legal profession. Federalist Society-affiliates leverage these connections to obtain prestigious clerkships, and powerful positions in academia and the executive, legislative, and judicial branches of federal and state government [3]. These members often stay connected to the Federalist Society network throughout their careers, which enables them to further promote the goals of the movement by helping younger lawyers follow similar career paths [5,10]. And, perhaps most importantly from our perspective, by maintaining these connections, the conservative goals of the Federalist Society are continually reinforced in the minds of affiliates, whose loyalty is rewarded as they move up through the conservative legal movement and in positions of power in government and the academy. In this sense, Federalist Society-affiliates learn that promoting the group’s interest is also a way of promoting their self-interest [14].

The third primary role of the Federalist Society – or more accurately its powerful leaders, affiliates, and members – involves advising high-ranking governmental actors [3,10,11]. This includes Federalist Society-affiliates in the executive, legislative, and judicial branches, who develop and promote policies consistent with the goals of the conservative legal movement. But the most important advising role for our purposes involves counseling presidents on federal judicial nominations, which represents perhaps the Federalist Society’s greatest successes [3,11]. Indeed, presidents George H.W. Bush, George W. Bush, and Donald Trump relied heavily on the advice of Federalist Society-affiliates to help select their federal judges [10,15]. Moreover, during his first term, Trump essentially outsourced the selection of his judicial nominees – including three Supreme Court justices – to Leonard Leo, the former Executive Vice President of the Federalist Society and current Co-Chair of the Board of Directors [2]. Importantly, the judges and justices with Federalist Society connections are presumed by presidents to be extremely conservative, and are arguably chosen as much for their Federalist Society connections as for any other qualification they possess [11,15].

The Federalist Society and its members and affiliates often interact across decades: first in law school, and later in the many networking opportunities the organization provides. Further, affiliates of the organization are often rewarded for their loyalty to the group through placements in elite clerkships, the legal academy, and in the private and public sector. During the course of these decades, the Federalist Society is able to instill and reinforce the core values of the organization in those affiliated with it. For Supreme Court justices affiliated with the Federalist Society, we believe this may result in two behavioral manifestations. First, they may be more conservative than their non-Federalist Society counterparts and, second, they may be more *consistently* conservative than non-Federalist Society-affiliated justices.

At first glance, it may not be surprising that Federalist Society-affiliated justices are expected to be conservative, considering that they were all nominated by Republican presidents. However, we argue that Federalist Society-affiliated justices are uniquely conservative. Such is the case because these justices have the conservative values of the Federalist Society instilled in them relatively early in their careers (although they may affiliate with the organization at any time in their careers), helping them more easily identify how legal disputes can be leveraged to advance the conservative agenda. This comes through in their commitments to originalism, the preferred judicial philosophy of the Federalist Society, which is often used to justify conservative outcomes [3]. Importantly, these conservative values are reinforced in the many Federalist Society networking events that they attend once on the bench. In addition, their affiliation with the Federalist Society acts as a cue to appointing presidents that they are a particular type of conservative [3]. To wit, since it was founded in 1982, there have been only three non-Federalist Society-affiliated nominees to the Supreme Court by Republican presidents: Anthony Kennedy (who was nominated only after Robert Bork, an early champion of the Federalist Society was rejected by the Senate, and who often found himself the Court’s swing voter), David Souter (who frequently ended up voting with the Court’s liberals), and Harriet Miers (who was withdrawn after Republicans worried about her conservative bona fides) [3,11,15]. Given this, we expect that Federalist Society-affiliated justices will be more likely to cast conservative votes than other justices, even after controlling for judicial ideology.

We also expect that Federalist Society-affiliated justices will be more ideologically consistent in their decision making than other justices. By this, we mean that they will seldom deviate from conservative ideological voting behavior, as compared to their counterparts who are not affiliated with the Federalist Society.

First, because Federalist Society-affiliates often interact with the organization early in their legal careers, this shapes their views of the law at a formative stage, which is then reinforced in further interactions with the organization and those in the conservative legal network. To put it differently, affiliation with the Federalist Society helps to make a justice a “true believer” in the organization’s goals, which are the goals of the conservative legal movement, and may lead to consistent conservative decision making [3,5,9].

Second, the desire for the acceptance and praise of their peers in the conservative legal movement may promote consistency in the decision making of Federalist Society-affiliated justices. As [15] contends, Supreme Court justices are not immune from the desire for acceptance from peers, who tend to be social and economic elites. For Federalist Society-affiliates, these are elites in the conservative legal movement, who frequently ask them to speak at Federalist Society conferences, law schools, and other prestigious events. If a conservative Supreme Court justice were to vote against the interests of the conservative legal movement, this could affect their standing in this legal community, and lead to public critique in their elite social circles. Indeed, members of the Federalist Society have spoken about their desire to keep justices “in check,” and their belief that they have succeeded in doing so [3].

Finally, and related, there are tangible benefits that come from remaining in the good graces of the conservative legal movement that may promote consistency in decision making. The aforementioned Federalist Society conferences are not simply a place for spirited debate, they are also an opportunity for justices to foster and make connections with wealthy elites [1,2]. Indeed, extensive reporting has revealed that Justices Alito and Thomas have financially benefited from these relationships in the form of lavish gifts and vacations [1]. The financial incentives for Federalist Society-affiliated justices to retain good relationships with the Federalist Society’s network are clear. While friendships may continue after an ideological divide, it is unlikely that a justice would receive these extensive tangible benefits if that justice voted against the interests of the wealthy elites in the Federalist Society network. For these reasons, we expect that Federalist Society-affiliated justices will be more ideologically consistent in their decision making than other justices.

## Materials and methods

To investigate whether justices affiliated with the Federalist Society are both more conservative and more ideologically consistent than their colleagues, we use data from *The Supreme Court Database* [16] and focus our analysis on the 1986–2022 terms. This corresponds to the period in which the first Federalist Society-affiliated justices joined the Court. The S1 Appendix contains information on other time periods, details on the coding of our control variables and statistical model, and the results of alternative model specifications, which corroborate the results presented here.

The unit of analysis is each justice’s vote in orally argued cases, and the dependent variable is the ideological direction of that vote, coded 1 for a conservative vote, and 0 for a liberal vote. Liberal votes are, for example, pro-civil rights or liberties claimant, pro-criminal defendant, pro-union, pro-environmental protection, pro-exercise of judicial power, and pro-federal government in disputes with states. Conservative votes are opposite thereof [16].

Because we have theoretical expectations for influences on both the mean and variance of the dependent variable (the latter of which captures consistency), we utilize a heteroskedastic probit model, and cluster our standard errors on case citation [17]. The heteroskedastic probit model differs from the traditional probit in that the heteroskedastic probit model allows researchers to model the variance based on predictor variables, while the traditional probit model assumes the variance to be constant. The heteroskedastic probit model estimates two equations: 1) a mean equation that captures influences on the ideological direction of the justices’ votes; and 2) a variance equation that captures influences on the ideological consistency of the justices’ voting behavior.

Our key independent variable is whether a justice is/was affiliated with the Federalist Society, scored 1 for justices Alito, Barrett, Gorsuch, Kavanaugh, Roberts, Scalia, and Thomas, and 0 for other justices [3,11]. In the mean equation, positive signs associated with variables indicate that they enhance the likelihood of observing a conservative vote. In the variance equation, positive signs indicate they increase the variance surrounding a conservative vote, and therefore contribute to more ideologically inconsistent voting behavior. Accordingly, we expect that the *Federalist Society* variable will be positively signed in the mean equation, and negatively signed in the variance equation.

## Results

**Fig 1:**
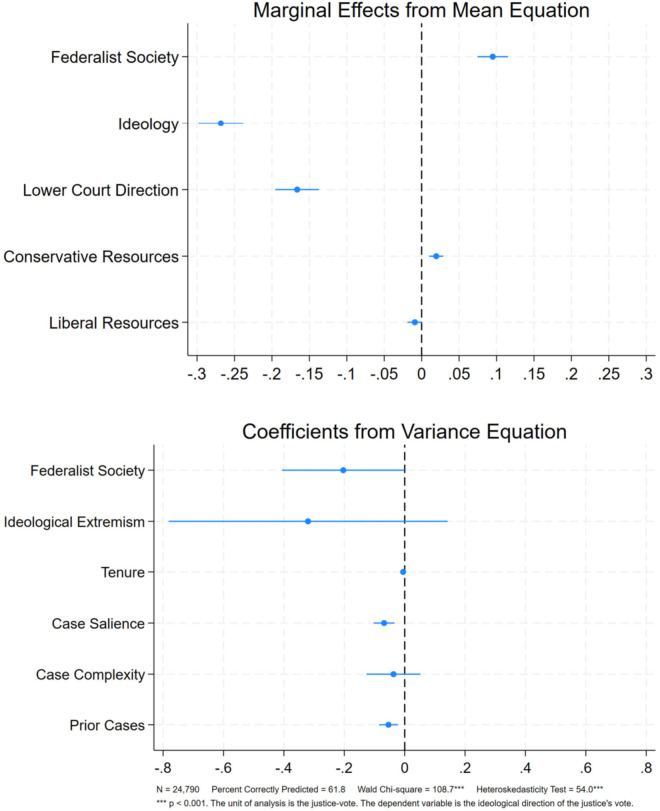
Heteroskedastic probit model of supreme court voting, 1986-2022.

Our results lend strong support for our theoretical expectations, demonstrating that Federalist Society-affiliated justices are both more conservative than their non-Federalist Society counterparts, and are more consistently ideologically conservative. This is shown in Fig 1, which reports the results of a heteroskedastic probit model of almost 25,000 votes in which the dependent variable is the ideological direction of the justice’s voting behavior in a case. The heteroskedasticity test indicates that we can reject the assumption of equal variance, indicating that the heteroskedastic probit model is superior to the homoscedastic probit model in terms of model fit (Heteroskedasticity Test = 54.0, p < 0.001).

Beginning with the mean equation (top figure), the marginal effects indicate that justices affiliated with the Federalist Society are about 9.5 percentage points more likely to cast a conservative vote, as compared to non-affiliated justices. This is a substantively large difference, and it is particularly notable that it holds even when accounting for each justice’s ideology. With respect to the *Ideology* variable, a one standard deviation increase in this variable – indicating that a justice is more liberal – results in about an 7 percentage point decrease in the likelihood of observing a conservative vote. Thus, the substantive difference of affiliating with the Federalist Society is a bit larger than a one-standard deviation change in judicial ideology.

The variance equation (bottom figure) allows for the examination of the variance surrounding a justice’s voting behavior; that is, the conditions under which voting behavior is more or less consistent [17]. Most significantly, this portion of the model indicates that Federalist Society-affiliated justices are more ideologically consistent in their decision making than other justices. Viewed in combination with the mean equation, this means that Federalist Society-affiliates are not only more conservative than their colleagues, they are also more *consistently* conservative. Since justices often cast thousands of votes over the course of their careers, this consistency is capable of steering American law in the conservative direction in substantial ways over long periods of time, suggesting the Court’s recent rightward turn will last for decades.

**Fig 2:**
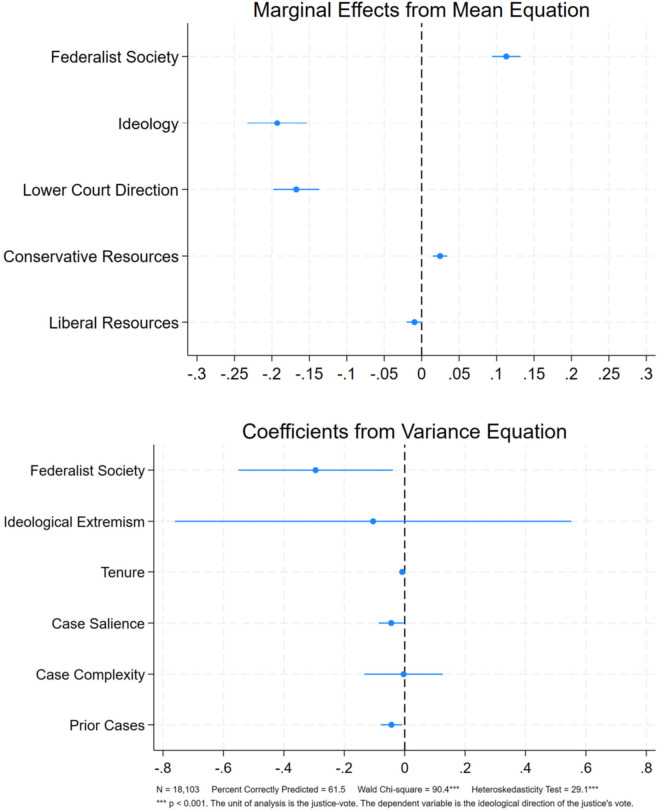
Heteroskedastic probit model of republican supreme court voting, 1986-2022.

To ensure the robustness of our results, in Fig 2, we report the results of the same heteroskedastic probit model, but limit the sample to include only justices appointed by Republican presidents. Our results hold. Compared to other Republican appointees, Federalist Society-affiliated justices are 11 percentage points more likely to cast conservative votes (mean equation), and are more ideologically consistent in their decision making (variance equation).

## Discussion and conclusions

The Supreme Court has taken a dramatic turn to the right in recent terms, fundamentally reshaping Americans’ lives in a variety of ways. In this article, we sought to investigate one possible explanation for this development: affiliation with the Federalist Society. Consistent with our expectations, we find that Federalist Society-affiliated justices are different: they are more conservative and more *consistently* conservative than their colleagues on the bench.

To the best of our knowledge, we are the first to unearth these important differences on the Supreme Court, which have significant implications. For instance, they help shed light on how the Federalist Society is capable of keeping its conservative affiliates from wandering from the movement’s goals by fostering consistency in their voting behavior. Our results also offer insight into the benefits that elites in the Federalist Society obtain when they provide justices with lavish trips and vacations: consistently conservative voting behavior. And, we believe our findings are likely to be generalizable to the behavior from the hundreds of Federalist Society-affiliated judges on the lower federal [4] and state courts. After all, like Supreme Court justices, lower court judges who are affiliated with the Federalist Society have the values of the conservative legal movement instilled in them relatively early in their careers, which are subsequently reinforced as they move up the ranks of the legal system [3,11]. This is clearly an important area for future research that will benefit by investigating how affiliation with the Federalist Society can shape not only judicial voting behavior, but also the consistency of that behavior. And, it will be important to examine the extent to which the behavior of Federalist Society-affiliated lower court judges is driven by ideological commitment, strategic efforts to secure promotion in the court system, or both. Indeed, demonstrating the consistent application of conservative decision making can act as a strong signal for judges seeking promotion [18]. Exploring this topic will also help clarify the causal mechanism(s) related to affiliation with the Federalist Society and consistent conservativism, a limitation of this article’s descriptive focus. Moreover, we believe that adapting the theoretical and methodological approaches used in this article to understand how similar training and networking can shape the behavior of other actors will prove very useful, especially for individuals operating in institutions structured to shield them from public and political pressures.

## Supporting information

S1 Appendix The Consistency of Federalist Society-Affiliated U.S. Supreme Court Justices. (DOCX)
