# Supplementary material for: The consistency of Federalist Society-affiliated U.S. supreme court justices
Source: PLoS One. 2025 Aug 18;20(8):e0329692. doi: 10.1371/journal.pone.0329692 (PMC12360514; doi:10.1371/journal.pone.0329692)
Supplement: S1 Appendix — (DOCX) [file pone.0329692.s001.docx]

**S1 Appendix**

**The Consistency of Federalist Society-Affiliated**

**U.S. Supreme Court Justices**

Tim Komatsu^1#^, Paul M. Collins, Jr.^12#*^

^1^Department of Political Science, University of Massachusetts Amherst, Amherst, Massachusetts, United States

^2^Department of Legal Studies, University of Massachusetts Amherst, Amherst, Massachusetts, United States

^#^These authors contributed equally to this work

* Corresponding author

Email: [pmcollins@umass.edu](mailto:pmcollins@umass.edu)

**The Heteroskedastic Probit Model**

Our theoretical argument is that justices affiliated with the Federalist Society will be both more conservative than their colleagues who are not affiliated with the Federalist Society, and will exhibit more consistent voting behavior than those colleagues. Accordingly, we need to employ a statistical model that allows us to model influences on both the mean of the justices’ voting behavior, which is common in political science research, and the variance of the justices’ voting behavior, which is a less common approach. To do this, we employ the heteroskedastic probit model, which political scientists have successfully used to investigate topics including judicial decision making (e.g., Collins 2008; Kaheny, Haire, and Benesh 2008), public attitudes toward abortion rights (Alvarez and Brehm 1995), voter choice (e.g., Box-Steffensmeier et al. 2015), strategic interaction between nations (Bas 2012), and much more. The heteroskedastic probit model differs from the traditional (homoskedastic) probit in that the heteroskedastic probit model allows researchers to model the variance based on predictor variables, while the traditional probit model assumes the variance to be constant (equal to one) (Greene 2012). In the current context, the heteroskedastic probit model estimates two equations: 1) a mean equation that captures influences on the ideological direction of the justices’ votes; and 2) a variance equation that captures influences on the ideological consistency of the justices’ voting behavior.

The heteroskedastic probit model also provides the results of a heteroskedasticity test, which is a likelihood ratio test evaluating model fit between the traditional (homoscedastic) probit model and the heteroskedastic probit model. As Figure 1 in the main text and Appendix Table 1 below reveal, we can reject the assumption of equal variance, thus indicating that the heteroskedastic probit model is superior to the homoscedastic probit model in terms of model fit (Heteroskedasticity Test = 54.0, p < 0.001).

**Operationalization of Control Variables**

In addition to including a variable indicating whether a justice was/is affiliated with the Federalist Society, we also include several control variables in the statistical model to account for other factors that influence judicial choice. In the mean equation (predicting the likelihood of a conservative vote), we include four control variables. *Ideology* captures each justice’s Segal and Cover (1989) score, which are assessments of judicial ideology based on newspaper editorials characterizing Supreme Court nominees as conservative or liberal. We obtained this information for all justices except Ketanji Brown Jackson from Epstein et al. (2022). We obtained Jackson’s Segal and Cover score from Boyd, Collins, and Ringhand (2023). This variable ranges from 0 to 1, with higher scores indicating more liberal ideologies. We expect this variable will be negatively signed, indicating that more liberal justices are less likely to cast conservative votes.

*Lower Court Direction* is scored 1 if the lower court decision was conservative in direction, and 0 if it was liberal in direction. This variable is included to account for the Supreme Court’s tendency to reverse lower court decisions (Segal and Spaeth 2002), and is based on information in Spaeth et al. (2024). We expect this variable will be negatively signed, indicating that a justice is less likely to cast a conservative vote if the lower court rendered a conservative decision.

Our final two variables in the mean equation capture litigant resources, which have the potential to shape judicial decision making because higher resourced litigants are more likely to come out ahead in litigation (Galanter 1974). Following Gleason and Smart (2023) each litigant is scored as follows: 1 = individuals, 2 = businesses, 3 = interest groups, 4 = state and local governments, 5 = federal government.^^[[1]](#footnote-1)^^ We expect that *Conservative Resources* will be positively signed, indicating that a justice is more likely to vote conservatively if the litigant arguing for the conservative position is ranked higher on the resource continuum. We expect that *Liberal Resources* will be negatively signed for the same reason.

We include five control variables in the variance equation. *Ideological Extremism* is intended to capture the extent to which a justice may have a relatively extreme ideology. To measure this, we take the absolute value of the justice’s Segal and Cover (1989) score after subtracting 0.5 from it. This variable ranges from 0 to 0.5, with higher values indicating that a justice has a more extreme conservative or liberal ideology. Accordingly, we expect that they will exhibit more consistent voting behavior, and therefore this variable will be negatively signed (e.g., Collins 2008).

*Tenure* is intended to capture the possibility that justices may go through an acclimation period on the Court, exhibiting more variable voting behavior earlier in their careers and becoming more consistent over time (e.g., Kaheny, Haire, and Benesh 2008). This variable is the number of terms each justice served on the Court. We expect it will be negatively signed, indicating that justices who have served for longer periods of time will be more stable in their decision making.

*Case Salience* is included to account for the possibility that the justices’ voting behavior may be more ideologically consistent in important cases (e.g., Collins 2008). The logic here is that the justices are likely to be especially engaged with salient cases, relying more on their ideological preferences in an effort to ensure those preferences are enacted into law (e.g., Unah and Hancock 2006). This should translate into more consistent ideological voting behavior compared to relatively routine disputes that may not be as ideologically significant for the justices. This variable is based on the Case Salience Index developed by Collins and Cooper (2012), updated by Scott, Lane, and Schoenherr (n.d.) and Cota et al. (2024) through the 2021 term. We updated the data through the 2022 term following the coding rules in Collins and Cooper (2012). Because higher scores on this variable indicate that a case is more salient, we expect it will be negatively signed, indicating that a justice will exhibit more consistent voting behavior in more significant cases.

*Case Complexity* is intended to capture the possibility that a justice’s voting behavior may be more variable in complex cases, owing to the nature of such cases. This variable is based on a factor analysis of the number of legal provisions and issues in a case, and the number of opinions in the case, following Maltzman, Spriggs, and Wahlbeck (2000, 47). We expect this variable will be positively signed, demonstrating that a justice’s voting behavior is more inconsistent in complex disputes.

Our final control variable in the variance equation captures the consistency that may develop over time as justices sit together on a Court with no membership change for long periods of time (e.g., Collins 2008). This variable is the number of cases each natural court decided prior to the case at hand during which there was no membership change, divided by 100 to make the presentation of the coefficient more manageable. We expect this variable will be negatively signed, indicating that a justice’s voting behavior will be more consistent as the number of cases decided by a natural court increases.

Appendix Table 1 reports the expected directions of the independent variables on the mean and/or variance of the dependent variable.

**Appendix Table 1. Expected Directions of the Independent Variables**

|  | **Mean of Dependent Variable** | **Variance of Dependent Variable** |
| --- | --- | --- |
| Federalist Society | + | - |
| **Control Variables in Mean Equation** |  |  |
| Ideology  Lower Court Direction  Conservative Resources  Liberal Resources | -  -  +  - |  |
|  |  |  |
| **Control Variables in Variance Equation**  Ideological Extremism  Tenure  Case Salience  Case Complexity  Prior Cases |  | -  -  -  +  - |
|  |  |  |

**Full Model Results and Interpretation**

Appendix Table 2 is a coefficient table that corresponds to Figure 1 in the main text. The model correctly predicts 61.8% of the justices’ votes, for a percent reduction in error of 23.1%. The heteroskedasticity test statistic indicates that the heteroskedastic probit model provides a better fit of the data than the standard (homoskedastic) probit model, which assumes equal variance.

Our results lend strong support for our theoretical expectations, demonstrating that Federalist Society-affiliated justices are both more conservative than their non-Federalist Society counterparts, and are more consistently ideologically conservative. Beginning with the mean equation, the results indicate that justices affiliated with the Federalist Society are about 9.5 percentage points more likely to cast a conservative vote, as compared to non-affiliated justices. This is a substantively large difference, and it is particularly notable that it holds even when controlling for each justice’s ideology. With respect to the *Ideology* variable, a one standard deviation increase in this variable – indicating that a justice is more liberal – results in about an 7 percentage point decrease in the likelihood of observing a conservative vote. Thus, the substantive effect of affiliating with the Federalist Society is a bit larger than a one-standard deviation change in judicial ideology.

**Appendix Table 2. Heteroskedastic Probit Model of Supreme Court Voting, 1986-2022**

|  | **Coefficient** | **Marginal Effect** |
| --- | --- | --- |
| ***Mean Equation*** |  |  |
| Federalist Society | 0.14*** | +9.5*** |
|  | (0.021) |  |
| Ideology | -0.43*** | -26.8*** |
|  | (0.051) |  |
| Lower Court Direction | -0.27*** | -16.6*** |
|  | (0.042) |  |
| Conservative Resources | 0.031*** | +1.9*** |
|  | (0.0087) |  |
| Liberal Resources | -0.015 | -1.0 |
|  | (0.0084) |  |
| Constant | 0.13** |  |
|  | (0.046) |  |
| ***Variance Equation*** |  |  |
| Federalist Society | -0.20* |  |
|  | (0.10) |  |
| Ideological Extremism | -0.32 |  |
|  | (0.24) |  |
| Tenure | -0.0052 |  |
|  | (0.0026) |  |
| Case Salience | -0.068*** |  |
|  | (0.018) |  |
| Case Complexity | -0.037 |  |
|  | (0.045) |  |
| Prior Cases | -0.054*** |  |
|  | (0.016) |  |
| N | 24790 |  |
| Percent Correctly Predicted | 61.8 |  |
| Wald Chi-square | 108.72*** |  |
| Heteroskedasticity Test | 54.0*** |  |
| * p<0.05, ** p<0.01, *** p<0.001 (one-tailed tests). Robust standard errors in parentheses. | | |
| The unit of analysis is the justice-vote. The dependent variable is whether or not the justice cast a conservative (1) or liberal vote (0). Marginal effect is a one-unit change in the variable of interest, holding all other variables at their mean or modal values, as appropriate. | | |

In their analysis of the voting behavior of Federalist Society-affiliated judges in nonconsensual states’ rights and search and seizure cases decided by the U.S. courts of appeals, Scherer and Miller (2009) find that Federalist Society-affiliated judges are more supportive of states’ rights and law enforcement than their non-affiliated counterparts, meaning that they vote more conservatively. Importantly, this finding holds even when they control for judicial ideology. Scherer and Miller (2009, 374) note that their evidence that Federalist Society-affiliated judges vote more conservatively may be due to their use of an imprecise measure of judicial ideology. We are less concerned about this possibility as our measure of judicial ideology – Segal and Cover (1989) scores – indicates that Federalist Society-affiliated justices are among the Court’s most conservative members. For instance, the Federalist Society-affiliated justices constitute 7 of the 11 most conservative justices on the Court, including 4 of the 5 most conservative justices. Their average Segal and Cover ideology score is 0.09, compared to 0.44 for non-affiliated justices (on a scale from 0 to 1, p < 0.001). Looking only at justices appointed by Republican presidents, Federalist Society-affiliated justices average score of 0.09, compared to 0.30 for non-affiliated conservatives (p < 0.001). Thus, it is evident that the Segal and Cover scores perform quite well measuring the ideology of Federalist Society-affiliated justices, meaning that our results suggest that Federalist Society-affiliated justices are different from even their Republican colleagues.^[[2]](#footnote-2)^

The model also reveals that a justice is about 17 percentage points more likely to vote liberally if the lower court handed down a conservative decision, which corroborates the Court’s well-known tendency to reverse lower court decisions. Litigant resources matter, too. For each one-unit increase on the resource continuum, a justice is about 2 percentage points more likely to vote in favor of the conservative litigant, and about 1 percentage point more likely to support the liberal litigant (p = 0.07, two-tailed).

The variance equation (bottom figure) allows for the examination of the variance surrounding a justice’s voting behavior; that is, the conditions under which voting behavior is more or less consistent (e.g., Collins 2008; Kaheny, Haire, and Benesh 2008). Most significantly, this portion of the model indicates that Federalist Society-affiliated justices are more ideologically consistent in their decision making than other justices. Viewed in combination with the mean equation, this means that Federalist Society-affiliates are not only more conservative than their colleagues, they are also more *consistently* conservative. Since justices often cast thousands of votes over the course of their careers, this consistency is capable of steering American law in the conservative direction in substantial ways over long periods of time, suggesting the Court’s recent rightward turn will last for decades.

The variance equation also indicates that justices are more ideologically consistent in salient cases. This suggests they rely more on their ideological preferences in such cases, resulting in more predictable voting behavior. Lastly, as the number of cases decided by a Court with no membership change increases, so too does the consistency of a justice’s voting behavior. This provides evidence that the justice learn about the preferences of their colleagues over time, resulting in more stable voting behavior. None of the other variables in the variance portion achieve statistical significance.

Appendix Table 3 reports the coefficients corresponding to Figure 2 in the text, corroborating the results discussed above when limiting the sample to only justices appointed by Republican presidents.

**Appendix Table 3. Heteroskedastic Probit Model of Republican Supreme Court Voting, 1986-2022**

|  | **Coefficient** | **Marginal Effect** |
| --- | --- | --- |
| ***Mean Equation*** |  |  |
| Federalist Society | 0.16*** | +11.2*** |
|  | (0.026) |  |
| Ideology | -0.34*** | -19.3*** |
|  | (0.043) |  |
| Lower Court Direction | -0.30*** | -16.7*** |
|  | (0.052) |  |
| Conservative Resources | 0.044*** | +2.4*** |
|  | (0.011) |  |
| Liberal Resources | -0.017 | -1.0 |
|  | (0.0098) |  |
| Constant | 0.094* |  |
|  | (0.047) |  |
| ***Variance Equation*** |  |  |
| Federalist Society | -0.30* |  |
|  | (0.13) |  |
| Ideological Extremism | -0.10 |  |
|  | (0.33) |  |
| Tenure | -0.0083* |  |
|  | (0.0042) |  |
| Case Salience | -0.045* |  |
|  | (0.021) |  |
| Case Complexity | -0.0037 |  |
|  | (0.066) |  |
| Prior Cases | -0.044* |  |
|  | (0.018) |  |
| N | 18103 |  |
| Percent Correctly Predicted | 61.5 |  |
| Wald Chi-square | 90.4*** |  |
| Heteroskedasticity Test | 29.1*** |  |
| * p<0.05, ** p<0.01, *** p<0.001 (one-tailed tests). Robust standard errors in parentheses. | | |
| The unit of analysis is the justice-vote. The dependent variable is whether or not the justice cast a conservative (1) or liberal vote (0). Marginal effect is a one-unit change in the variable of interest, holding all other variables at their mean or modal values, as appropriate. | | |

**Alternative Model Specifications**

**Correlation Among Independent Variables**

The strongest correlations among the independent variables in the models are between the *Federalist Society* and *Ideology* variables (r = 0.59), and the *Federalist Society* and *Ideological Extremism* variables (r = 0.57). This is to be expected. As discussed in the main text, Federalist Society-affiliated justices are among the most conservative justices on the Court, and are therefore also some of the most ideologically extreme justices on the Court.

To ensure that these correlations are not artificially driving the results, Appendix Table 4 reports the results of a heteroskedastic probit model that removes the *Ideology* and *Ideological Extremism* variables. This table corroborates the results in the main text, showing that Federalist Society-affiliated justices are both more conservative and more consistently conservative than non-affiliates. The main difference is that the marginal effect of the *Federalist Society* variable is much larger in the mean equation of Appendix Table 4, which makes sense since this variable is tapping into some justices’ conservative ideologies since this model no longer controls for ideology.

**Appendix Table 4. Heteroskedastic Probit Model of Supreme Court Voting without Ideology Variables, 1986-2022**

|  | **Coefficient** | **Marginal Effect** |
| --- | --- | --- |
| ***Mean Equation*** |  |  |
| Federalist Society | 0.29*** | +16.6*** |
|  | (0.030) |  |
| Lower Court Direction | -0.31*** | -16.5*** |
|  | (0.043) |  |
| Conservative Resources | 0.038*** | +2.0*** |
|  | (0.0096) |  |
| Liberal Resources | -0.017 | -0.9 |
|  | (0.0095) |  |
| Constant | -0.050 |  |
|  | (0.043) |  |
| ***Variance Equation*** |  |  |
| Federalist Society | -0.37*** |  |
|  | (0.093) |  |
| Tenure | -0.0026 |  |
|  | (0.0029) |  |
| Case Salience | -0.060** |  |
|  | (0.020) |  |
| Case Complexity | 0.051 |  |
|  | (0.072) |  |
| Prior Cases | -0.050** |  |
|  | (0.019) |  |
| N | 24790 |  |
| Percent Correctly Predicted | 60.6 |  |
| Wald Chi-square | 102.7*** |  |
| Heteroskedasticity Test | 45.15*** |  |
| * p<0.05, ** p<0.01, *** p<0.001 (one-tailed tests). Robust standard errors in parentheses. | | |
| The unit of analysis is the justice-vote. The dependent variable is whether or not the justice cast a conservative (1) or liberal vote (0). Marginal effect is a one-unit change in the variable of interest, holding all other variables at their mean or modal values, as appropriate. | | |

**Changing the Time Frame for Analysis**

In the main text, we examine the extent to which affiliation with the Federalist Society might influence judicial decision making by examining the justices’ votes during the 1986-2022 time frame, which corresponds to the time period in which the first Federalist Society-affiliate (Antonin Scalia) joined the Court to the last term for which data is available. We believe this is a useful time frame because it allows us to examine the difference between Federalist Society-affiliates and other justices during the time period in which they served together.

An alternative is to use data from the entire era of the modern Supreme Court, 1953-2022. Appendix Table 5 reports this information. These results corroborate those in the main text. That is, they reveal that Federalist Society-affiliated justices are about 6 percentage points more likely to cast conservative votes than their colleagues, and they are also more ideologically consistent in their voting behavior.

**Appendix Table 5. Heteroskedastic Probit Model of Supreme Court Voting, 1953-2022**

|  | **Coefficient** | **Marginal Effect** |  |
| --- | --- | --- | --- |
| ***Mean Equation*** |  |  |  |
| **Federalist Society** | 0.084*** | +6.0*** |  |
|  | (0.024) |  |  |
| **Ideology** | -0.58*** | -29.6*** |  |
|  | (0.050) |  |  |
| **Lower Court Direction** | -0.34*** | -17.2*** |  |
|  | (0.034) |  |  |
| **Conservative Resources** | 0.060*** | +3.0*** |  |
|  | (0.0083) |  |  |
| **Liberal Resources** | -0.022** | -1.1* |  |
|  | (0.0071) |  |  |
| **Constant** | 0.21*** |  |  |
|  | (0.043) |  |  |
| ***Variance Equation*** |  |  |  |
| **Federalist Society** | -0.22** |  |  |
|  | (0.083) |  |  |
| **Ideological Extremism** | -0.035 |  |  |
|  | (0.12) |  |  |
| **Tenure** | -0.0060*** |  |  |
|  | (0.0018) |  |  |
| **Case Salience** | -0.038** |  |  |
|  | (0.012) |  |  |
| **Case Complexity** | 0.011 |  |  |
|  | (0.030) |  |  |
| **Prior Cases** | -0.030** |  |  |
|  | (0.0098) |  |  |
| **N** | 55,912 |  |  |
| **Percent Correctly Predicted** | 63.9 |  |  |
| **Wald Chi-square** | 177.88*** |  |  |
| **Heteroskedasticity Test** | 35.75*** |  |  |
| * p<0.05, ** p<0.01, *** p<0.001 (one-tailed tests). Robust standard errors in parentheses. | | | |
| The unit of analysis is the justice-vote. The dependent variable is whether or not the justice cast a conservative (1) or liberal vote (0). Marginal effect is a one-unit change in the variable of interest, holding all other variables at their mean or modal values, as appropriate.  Another alternative is to use data from 1982-2022, which corresponds to the founding of the Federalist Society in 1982. This is a potentially useful time frame because any justice could have affiliated with the Federalist Society starting in 1982. Accordingly, this allows us to examine differences in the voting behavior of Federalist Society affiliates with others who could have, but opted not to, affiliate with the organization.^[[3]](#footnote-3)^ Appendix Table 6 reports this model. These results corroborate those in the main text. That is, they reveal that Federalist Society-affiliated justices are about 9 percentage points more likely to cast conservative votes than their colleagues, and they are also more ideologically consistent in their voting behavior.  **Appendix Table 6. Heteroskedastic Probit Model of Supreme Court Voting, 1982-2022**   \|  \| **Coefficient** \| **Marginal Effect** \| \| --- \| --- \| --- \| \| ***Mean Equation*** \|  \|  \| \| **Federalist Society** \| 0.14*** \| +8.9*** \| \|  \| (0.023) \|  \| \| **Ideology** \| -0.53*** \| -29.7*** \| \|  \| (0.056) \|  \| \| **Lower Court Direction** \| -0.33*** \| -18.4*** \| \|  \| (0.044) \|  \| \| **Conservative Resources** \| 0.047*** \| +2.6*** \| \|  \| (0.0097) \|  \| \| **Liberal Resources** \| -0.020* \| -1.1* \| \|  \| (0.0092) \|  \| \| **Constant** \| 0.17*** \|  \| \|  \| (0.052) \|  \| \| ***Variance Equation*** \|  \|  \| \| **Federalist Society** \| -0.19* \|  \| \|  \| (0.093) \|  \| \| **Ideological Extremism** \| -0.20 \|  \| \|  \| (0.20) \|  \| \| **Tenure** \| -0.0023 \|  \| \|  \| (0.0026) \|  \| \| **Case Salience** \| -0.035* \|  \| \|  \| (0.016) \|  \| \| **Case Complexity** \| -0.055 \|  \| \|  \| (0.042) \|  \| \| **Prior Cases** \| -0.048*** \|  \| \|  \| (0.014) \|  \| \| **N** \| 29,720 \|  \| \| **Percent Correctly Predicted** \| 62.7 \|  \| \| **Wald Chi-square** \| 137.84*** \|  \| \| **Heteroskedasticity Test** \| 40.24*** \|  \| \| * p<0.05, ** p<0.01, *** p<0.001 (one-tailed tests). Robust standard errors in parentheses. \| \| \| \| \| The unit of analysis is the justice-vote. The dependent variable is whether or not the justice cast a conservative (1) or liberal vote (0). Marginal effect is a one-unit change in the variable of interest, holding all other variables at their mean or modal values, as appropriate. \| \| \| \|  \| **Republican Appointees versus Federalist Society-Affiliates**  As we note in the main text, all Federalist Society-affiliated justices were appointed by Republican presidents. As a result, its possible (though we believe unlikely) that the variable indicating affiliation with the Federalist Society is primarily serving as an indicator variable for justices appointed by Republican presidents. To examine this more closely, Appendix Table 7 replaces the *Federalist Society* variable for a variable indicating a justice was a *Republican Appointee*, scored 1 if a Republican president appointed the justice, and 0 otherwise. As this table indicates, the *Republican Appointee* variable fails to achieve statistical significance in both the mean and variance equations. This indicates that justices nominated by Republican presidents are not more conservative or more consistently conservative then their Democratic counterparts when ideology is controlled for. Conversely, the results in the main text indicate that justices affiliated with the Federalist Society are more conservative and more consistently conservative than non-Federalist Society-affiliated justices, even when ideology is controlled for.  **Appendix Table 7. Heteroskedastic Probit Model of Supreme Court Voting with Republican Indicator Variable, 1986-2022**   \|  \| **Coefficient** \| **Marginal Effect** \| \| --- \| --- \| --- \| \| ***Mean Equation*** \|  \|  \| \| Republican Appointee \| 0.033 \| Not significant \| \|  \| (0.025) \|  \| \| Ideology \| -0.57*** \| -35.3*** \| \|  \| (0.083) \|  \| \| Lower Court Direction \| -0.27*** \| -17.0*** \| \|  \| (0.052) \|  \| \| Conservative Resources \| 0.032*** \| +2.0*** \| \|  \| (0.0091) \|  \| \| Liberal Resources \| -0.017* \| -1.0* \| \|  \| (0.0092) \|  \| \| Constant \| 0.21** \|  \| \|  \| (0.074) \|  \| \| ***Variance Equation*** \|  \|  \| \| Republican Appointee \| -0.095 \|  \| \|  \| (0.088) \|  \| \| Ideological Extremism \| -0.80*** \|  \| \|  \| (0.23) \|  \| \| Tenure \| 0.0085** \|  \| \|  \| (0.0030) \|  \| \| Case Salience \| -0.057** \|  \| \|  \| (0.018) \|  \| \| Case Complexity \| -0.026 \|  \| \|  \| (0.048) \|  \| \| Prior Cases \| -0.063*** \|  \| \|  \| (0.017) \|  \| \| N \| 24790 \|  \| \| Percent Correctly Predicted \| 62.1 \|  \| \| Wald Chi-square \| 95.12*** \|  \| \| Heteroskedasticity Test \| 37.82*** \|  \| \| * p<0.05, ** p<0.01, *** p<0.001 (one-tailed tests). Robust standard errors in parentheses. \| \| \| \| The unit of analysis is the justice-vote. The dependent variable is whether or not the justice cast a conservative (1) or liberal vote (0). Marginal effect is a one-unit change in the variable of interest, holding all other variables at their mean or modal values, as appropriate. \| \| \|  \| **Including Term Dummies**  In the main text, we run the heteroskedastic probit model without including indicator variables for each term. Appendix Table 8 report the results inclusive of dummy variables for each term, save one, to account for the possibility that some temporal dynamic might shape the results. As this table indicates, the results are generally robust to the inclusion of temporal dummy variables. That is, Federalist Society-affiliated justices are about 10 percentage points more likely to cast a conservative vote, and are also more ideologically consistent than their non-Federalist Society-affiliated counterparts (p = 0.09, two-tailed in the variance equation). Note that none of the dummy variables achieve statistical significance (the closest is the 2009 indicator variable, which is significant at p = 0.13, two-tailed), suggesting these variables are not essential to include in the statistical model. This is also corroborated by the difference in the Bayesian information criterion (BIC) between the model without term dummies (BIC = 32,363.69) and the model with term dummies (BIC = 32,607.26).  **Appendix Table 8. Heteroskedastic Probit Model of Supreme Court Voting with Term Dummies, 1986-2022**   \|  \| **Coefficient** \| **Marginal Effect** \| \| --- \| --- \| --- \| \| ***Mean Equation*** \|  \|  \| \| Federalist Society \| 0.15*** \| +10.4*** \| \|  \| (0.023) \|  \| \| Ideology \| -0.43*** \| -26.7*** \| \|  \| (0.050) \|  \| \| Lower Court Direction \| -0.27*** \| -17.0*** \| \|  \| (0.046) \|  \| \| Conservative Resources \| 0.031*** \| +2.0*** \| \|  \| (0.0091) \|  \| \| Liberal Resources \| -0.015 \| -0.9 \| \|  \| (0.0088) \|  \| \| Constant \| 0.13* \|  \| \|  \| (0.062) \|  \| \| ***Variance Equation*** \|  \|  \| \| Federalist Society \| -0.19 \|  \| \|  \| (0.11) \|  \| \| Ideological Extremism \| -0.29 \|  \| \|  \| (0.26) \|  \| \| Tenure \| -0.0065** \|  \| \|  \| (0.0024) \|  \| \| Case Salience \| -0.067** \|  \| \|  \| (0.021) \|  \| \| Case Complexity \| -0.017 \|  \| \|  \| (0.053) \|  \| \| Prior Cases \| -0.052** \|  \| \|  \| (0.016) \|  \| \| N \| 24790 \|  \| \| Percent Correctly Predicted \| 62.4 \|  \| \| Wald Chi-square \| 122.72*** \|  \| \| Heteroskedasticity Test \| 40.39*** \|  \| \| * p<0.05, ** p<0.01, *** p<0.001 (one-tailed tests). Robust standard errors in parentheses. \| \| \| \| The unit of analysis is the justice-vote. The dependent variable is whether or not the justice cast a conservative (1) or liberal vote (0). Marginal effect is a one-unit change in the variable of interest, holding all other variables at their mean or modal values, as appropriate. \| \| \| \| Model includes 36 term dummy variables, none of which are statistically significant at p<0.05. \| \| \| \|  \| \| \| \| \| --- \| --- \| --- \| --- \| --- \| --- \| --- \| --- \| --- \| --- \| --- \| --- \| --- \| --- \| --- \| --- \| --- \| --- \| --- \| --- \| --- \| --- \| --- \| --- \| --- \| --- \| --- \| --- \| --- \| --- \| --- \| --- \| --- \| --- \| --- \| --- \| --- \| --- \| --- \| --- \| --- \| --- \| --- \| --- \| --- \| --- \| --- \| --- \| --- \| --- \| --- \| --- \| --- \| --- \| --- \| --- \| --- \| --- \| --- \| --- \| --- \| --- \| --- \| --- \| --- \| --- \| --- \| --- \| --- \| --- \| --- \| --- \| --- \| --- \| --- \| --- \| --- \| --- \| --- \| --- \| --- \| --- \| --- \| --- \| --- \| --- \| --- \| --- \| --- \| --- \| --- \| --- \| --- \| --- \| --- \| --- \| --- \| --- \| --- \| --- \| --- \| --- \| --- \| --- \| --- \| --- \| \| \| --- \| --- \| --- \| --- \| --- \| --- \| --- \| --- \| --- \| --- \| --- \| --- \| --- \| --- \| --- \| --- \| --- \| --- \| --- \| --- \| --- \| --- \| --- \| --- \| --- \| --- \| --- \| --- \| --- \| --- \| --- \| --- \| --- \| --- \| --- \| --- \| --- \| --- \| --- \| --- \| --- \| --- \| --- \| --- \| --- \| --- \| --- \| --- \| --- \| --- \| --- \| --- \| --- \| --- \| --- \| --- \| --- \| --- \| --- \| --- \| --- \| --- \| --- \| --- \| --- \| --- \| --- \| --- \| --- \| --- \| --- \| --- \| --- \| --- \| --- \| --- \| --- \| --- \| --- \| --- \| --- \| --- \| --- \| --- \| --- \| --- \| --- \| --- \| --- \| --- \| --- \| --- \| --- \| --- \| --- \| --- \| --- \| --- \| --- \| --- \| --- \| --- \| --- \| --- \| --- \| --- \| --- \| --- \| --- \| --- \| --- \| --- \| --- \| --- \| --- \| --- \| --- \| --- \| --- \| --- \| --- \| --- \| --- \| --- \| --- \| --- \| --- \| --- \| --- \| --- \| --- \| --- \| --- \| --- \| --- \| --- \| --- \| --- \| --- \| --- \| --- \| --- \| --- \| --- \| --- \| --- \| --- \| --- \| --- \| --- \| --- \| --- \| --- \| --- \| --- \| --- \| --- \| --- \| --- \| --- \| --- \| --- \| --- \| --- \| --- \| --- \| --- \| --- \| --- \| --- \| --- \| --- \| --- \| --- \| --- \| --- \| --- \| --- \| --- \| --- \| --- \| --- \| --- \| --- \| --- \| --- \| --- \| --- \| --- \| --- \| --- \| --- \| --- \| --- \| --- \| --- \| --- \| --- \| --- \| --- \| --- \| --- \| --- \| --- \| --- \| --- \| \|  \| | | | |

**Homoskedastic Probit Model**

For those interested, Appendix Table 9 reports the results of the homoscedastic probit model that assumes equal variance. Although this model has an inferior model fit than the heteroskedastic probit model (as indicated in the heteroskedasticity test in Appendix Table 1), it corroborates that, even when controlling for judicial ideology, Federalist Society-affiliated justices are about 8 percentage points more likely to cast conservative votes than their non-Federalist Society colleagues. Because this model assumes equal variance, it cannot provide insight into whether Federalist Society-affiliated justices are more consistent than their unaffiliated colleagues.

**Appendix Table 9. Homoskedastic Probit Model** **of Supreme Court Voting, 1986-2022**

|  | **Coefficient** | **Marginal Effect** |
| --- | --- | --- |
| ***Mean Equation*** | 0.25*** |  |
| Federalist Society | (0.028) | +9.8*** |
|  | -0.70*** |  |
| Ideology | (0.042) | -27.4*** |
|  | -0.48*** |  |
| Lower Court Direction | (0.036) | +18.9*** |
|  | 0.055*** |  |
| Conservative Resources | (0.014) | +2.2*** |
|  | -0.032* |  |
| Liberal Resources | (0.015) | -1.2* |
|  | 0.28*** |  |
| Constant | (0.070) |  |
| N | 24790 |  |
| Percent Correctly Predicted | 62.4 |  |
| Wald Chi-square | 122.72*** |  |
| * p<0.05, ** p<0.01, *** p<0.001 (one-tailed tests). Robust standard errors in parentheses. | | |
| The unit of analysis is the justice-vote. The dependent variable is whether or not the justice cast a conservative (1) or liberal vote (0). Marginal effect is a one-unit change in the variable of interest, holding all other variables at their mean or modal values, as appropriate. | | |

**Model with Count of Federalist Society-affiliated Justices**

In Appendix Table 10, we investigate the extent to which the number of Federalist Society-affiliated justices serving together may influence the voting behavior of Federalist Society-affiliates. The idea is that justices may be more likely to cast conservative votes as the number of Federalist Society-affiliates on the Court increases. To explore this possibility, we include a variable indicating the number of Federalist Society-affiliated justices serving on the Court (*Number of Federalist Society*) As Appendix Table 9 reveals, this variable does not achieve statistical significance (p = 0.18, two-tailed).^[[4]](#footnote-4)^ The Bayesian information criterion (BIC) indicates that the model with the *Number of Federalist Society* justices (BIC = 32,366.6) is less preferrable to the model without this variable (BIC = 32,363.69).

**Appendix Table 10. Heteroskedastic Probit Model** **of Supreme Court Voting with Number of Federalist Society Justices Variable, 1986-2022**

|  | **Coefficient** | **Marginal Effect** |
| --- | --- | --- |
| ***Mean Equation*** |  |  |
| Federalist Society | 0.16*** | +10.7*** |
|  | (0.023) |  |
| Number of Federalist Society | -0.010 | -0.7 |
|  | (0.0078) |  |
| Ideology | -0.41*** | -25.9*** |
|  | (0.047) |  |
| Lower Court Direction | -0.27*** | -16.6*** |
|  | (0.043) |  |
| Conservative Resources | 0.031*** | +1.9*** |
|  | (0.0087) |  |
| Liberal Resources | -0.014 | -1.0 |
|  | (0.0085) |  |
| Constant | 0.15** |  |
|  | (0.051) |  |
| ***Variance Equation*** |  |  |
| Federalist Society | -0.20 |  |
|  | (0.11) |  |
| Ideological Extremism | -0.25 |  |
|  | (0.25) |  |
| Tenure | -0.0071** |  |
|  | (0.0026) |  |
| Case Salience | -0.070*** |  |
|  | (0.018) |  |
| Case Complexity | -0.040 |  |
|  | (0.045) |  |
| Prior Cases | -0.049** |  |
|  | (0.016) |  |
| N | 24790 |  |
| Percent Correctly Predicted | 61.8 |  |
| Wald Chi-square | 109.38*** |  |
| Heteroskedasticity Test | 54.77*** |  |
| * p<0.05, ** p<0.01, *** p<0.001 (one-tailed tests). Robust standard errors in parentheses. | | |
| The unit of analysis is the justice-vote. The dependent variable is whether or not the justice cast a conservative (1) or liberal vote (0). Marginal effect is a one-unit change in the variable of interest, holding all other variables at their mean or modal values, as appropriate. | | |

**References Not in Main Text**

Alvarez, R. Michael, and John Brehm. 1995. “American Ambivalence Towards Abortion Policy: Development of a Heteroskedastic Probit Model of Competing Values.” *American Journal of Political Science* 39(4): 1055-1082.

Bas, Muhammet A. 2023. “Measuring Uncertainty in International Relations: Heteroskedastic Strategic Models.” *Conflict Management and Peace Science* 29(5): 490-520.

Boyd, Christina L., Paul M. Collins, Jr., and Lori A. Ringhand. 2023. *Supreme Bias: Gender and Race in U.S. Supreme Court Confirmation Hearings.* Stanford, CA: Stanford University Press.

Box-Steffensmeier, Janet, Micah Dillard, David Kimball, and William Massengill. 2015. “The Long and Short of It: The Unpredictability of Late Deciding Voters.” *Electoral Studies* 39: 181-194.

Collins Todd A., Cooper Christopher A. 2012. “Case Salience and Media Coverage of Supreme Court Decisions: Toward a New Measure.” *Political Research Quarterly* 65(2): 396-407.

Cota, Matthew T., Rachael B. Houston, Elizabeth A. Lane, and Jessica A. Schoenherr. 2024. “All the News That’s Fit to Not Print: Changing of Covering the United States Supreme Court.” Typescript.

Epstein, Lee, Thomas G. Walker, Nancy Staudt, Scott Hendrickson, and Jason Roberts. 2022. “The U.S. Supreme Court Justices Database.” <https://epstein.wustl.edu/justicesdata> (accessed June 3, 2024).

Galanter, Marc. 1974. ‘‘Why the ‘Haves’ Come Out Ahead: Speculations on the Limits of Legal Change.’’ *Law & Society Review* 9(1): 95-160

Greene, William H. 2012. *Econometric Analysis.* 7th ed. Upper Saddle River, NJ: Prentice Hall.

Kaheny, Erin B., Susan Brodie Haire, and Sara C. Benesh. 2008. “Change Over Tenure: Voting, Variance, and Decision Making on the US Courts of Appeals.” *American Journal of Political Science* 52(3): 490-503.

Maltzman, Forrest, James F. Spriggs II, and Paul J. Wahlbeck. 2000. *Crafting Law on the Supreme Court*. New York: Cambridge University Press.

Martin, Andrew D., and Kevin M. Quinn. 2002. “Dynamic Ideal Point Estimation via Markov Chain Monte Carlo for the U.S. Supreme Court, 1953-1999.” *Political Analysis* 10(2): 134–153.

Martin, Andrew D., and Kevin M. Quinn. 2005. “Can Ideal Point Estimates be Used as Explanatory Variables?” Typescript. <http://mqscores.wustl.edu/media/resnote.pdf>.

Scott, Jamil S., Elizabeth A. Lane, and Jessica A. Schoenherr. N.d. “You Better Shop Around: Litigant Characteristics and Support for Supreme Court Decisions.” *Journal of Politics* forthcoming.

Segal, Jeffrey A., and Albert D. Cover. 1989. “Ideological Values and the Votes of U.S. Supreme Court Justices.” *American Political Science Review* 83(2): 557-65.

Segal, Jeffrey A., and Harold J. Spaeth. 2002. *The Supreme Court and the Attitudinal Model Revisited.* New York: Cambridge University Press.

Unah, Isaac, and Ange-Marie Hancock. 2006. ‘‘U.S. Supreme Court Decision Making, Case Salience, and the Attitudinal Model.’’ *Law and Policy* 28(3): 295–320

1. The small number of litigants not fitting into these categories were assigned the mean score for the petitioner or respondent, as appropriate. [↑](#footnote-ref-1)
2. The Martin and Quinn (2002) scores are another widely used measure of judicial ideology. We forgo employing these scores since they are based on the votes we are interested in analyzing (i.e., their use would be using votes to predict votes). As Martin and Quinn (2005, 5) note, “if the focus of the study is votes on the merits on all cases [as is the case here], using Martin-Quinn scores is inappropriate.” [↑](#footnote-ref-2)
3. No justice joined the Federalist Society while serving on the Court; they all joined prior to their service on the Court. [↑](#footnote-ref-3)
4. The Federalist Society variable falls slightly out of statistical significance in the model with the Number of Federalist Society variable (p = 0.07, one-tailed). This is likely due to the introduction of multicollinearity in that the *Number of Federalist Society* variable is directly related to the *Federalist Society* variable. [↑](#footnote-ref-4)
